# The influence of the BDNF Val66Met genotype on emotional recognition memory in post-traumatic stress disorder

**DOI:** 10.1038/s41598-023-30787-6

**Published:** 2023-03-28

**Authors:** Emma Louise Nicholson, Michael I. Garry, Luke J. Ney, Chia-Ming K. Hsu, Daniel V. Zuj, Kim L. Felmingham

**Affiliations:** 1 Melbourne School of Psychological Sciences, University of Melbourne, Redmond Barry Building, Parkville, VIC 3010 Australia; 2 School of Psychological Sciences, University of Tasmania, Hobart, Australia; 3 Faculty of Health, School of Psychology and Counselling, Queensland University of Technology, Brisbane, Australia; 4 Experimental Psychopathology Lab, Department of Psychology, Swansea University, Swansea, UK

**Keywords:** Neuroscience, Psychology

## Abstract

Dysregulated consolidation of emotional memories is a core feature of posttraumatic stress disorder (PTSD). Brain Derived Neurotrophic Factor (BDNF) influences synaptic plasticity and emotional memory consolidation. The BDNF Val66Met polymorphism has been associated with PTSD risk and memory deficits respectively, although findings have been inconsistent, potentially due to a failure to control for important confounds such as sex, ethnicity, and the timing/extent of previous trauma experiences. Furthermore, very little research has examined the impact of BDNF genotypes on emotional memory in PTSD populations. This study investigated the interaction effects of Val66Met and PTSD symptomatology in an emotional recognition memory task in 234 participants divided into healthy control (*n* = 85), trauma exposed (TE: *n* = 105) and PTSD (*n* = 44) groups. Key findings revealed impaired negative recognition memory in PTSD compared to control and TE groups and in participants with the Val/Met compared to the Val/Val genotype. There was a group × genotype interaction showing no Met effect in the TE group despite significant effects in PTSD and controls. Results suggest that people previously exposed to trauma who do not develop PTSD may be protected from the BDNF Met effect, however more research is needed to replicate findings and to explore the epigenetic and neural processes involved.

## Introduction

Posttraumatic Stress Disorder (PTSD) is a psychiatric condition that affects 6.4% of the Australian population^1^ and is associated with significant distress and impairment in function and quality of life^2^. PTSD is characterised by re-experiencing symptoms (e.g. intrusive memories, flashbacks of the trauma), negative alterations in cognitions and mood, avoidance and hyper-arousal^3^. A central feature of PTSD is memory dysfunction, with over-consolidation of trauma memories into long term memory storage proposed as a core mechanism underlying PTSD^4,5^. This can result in negative intrusive memories, flashbacks and dreams of the trauma but also a tendency to recall or recognise trauma-related material better than neutral material^6^. PTSD has been characterised by cognitive changes in *involuntary* memory (no conscious initiation of the retrieval process) for a traumatic incident reflected in intrusive memories^3^, however it is also crucial to understand how PTSD may affect the *voluntary* (conscious initiation) retrieval of emotional experiences. Recognition memory is a key form of voluntary retrieval that has received comparatively little investigation in PTSD.

Recognition memory is the ability to identify a situation or stimulus that has been experienced previously^7^. It is known to involve two separate processes: recollection and familiarity. Recollection requires the conscious retrieval of the specific details of a past experience whereas familiarity is a faster process, resulting in simply knowing the item was presented without retrieving any associated contextual details^7,8^. Both recognition memory processes are associated with neural activation in the medial temporal lobe including the hippocampus and extra-hippocampal structures such as the perirhinal cortex^8,9^. Although much empirical research provides support for memory dysregulation in PTSD^4,10–13^, there is relatively less evidence for a specific deficit in recognition memory in PTSD populations and findings are mixed. In recognition memory studies with non-emotional stimuli, some evidence has been found for deficits in patients with PTSD compared to controls^14–16^ whereas others have found no differences^17,18^. In their systematic review of emotional memory in PTSD, Durand et al.^6^ acknowledged there are few studies examining emotional memory recognition in PTSD, with those that exist being highly heterogenous in terms of their methods and results. Most of these studies have found no differences between PTSD and controls in positive and neutral recognition memory^19–25^. Results for negative emotional recognition memory are again inconsistent due to methodological differences in the studies in terms of measurement, types of stimuli used (e.g. words or images) and characteristics of participants involved (e.g. sex, trauma exposure). Some studies have found better negative recognition memory in PTSD patients compared to controls^26,27^ whereas others have found a memory deficit^28^ or no differences^29,30^.

Increasing animal and human evidence suggests that the neurotrophin Brain Derived Neurotrophic Factor (BDNF) has a powerful influence on memory. BDNF is known to influence synaptic plasticity and long-term potentiation (LTP) which are essential for memory consolidation^31–33^. Further, BDNF expression in neural fear circuitry (amygdala, prefrontal cortex and hippocampus) is important for emotional memory formation, including fear memories^34,35^. In humans, a single nucleotide polymorphism (SNP) in the gene encoding BDNF results in the substitution of amino acid Valine (Val) for Methionine (Met) at position 66 (Val66Met) in the BDNF gene^36^. This Val66Met polymorphism or Met allele (Met/Met or Val/Met genotype) results in reduced activity-dependent secretion of BDNF and has been associated with reduced episodic memory and hippocampal function^37,38^.

Furthermore, research suggests this Met allele may be associated with increased risk of PTSD^39–41^. A recent study with over 1000 U.S. military veterans found increased risk and greater severity of PTSD in participants with the Met allele^42^, however other studies have not found these effects^43–45^. A meta-analysis^46^ found no overall effect of the Met allele on PTSD risk in the overall analysis however there was high heterogeneity amongst the samples. Further sub-analyses revealed there was a significant effect of the Met allele on PTSD risk when only PTSD and TE groups were compared, but no effect of the Met allele in the comparison between PTSD and healthy controls. This suggests that previous trauma exposure may be a significant moderator of the BDNF effect and therefore an important factor to control in BDNF research. Furthermore, as noted by the authors, research suggests that BDNF effects are influenced by sex (greater susceptibility in females)^39,47^ ethnicity^48^, and BMI^49,50^. Key recommendations for further research highlighted from this meta-analysis included the importance of controlling for variables such as sex, ethnicity, BMI and levels of trauma exposure which could explain much of the variability found in this literature.

Evidence from animal and human studies support the influence of BDNF on memory and neurological function. In animal models, infusion of BDNF antibodies prior to training resulted in impaired learning in rats^51^ as well as impaired memory and inhibitory avoidance^52^. In studies using genetic rodent models, insertion of the Met allele (BDNF ‘knock in’ mice, see^36^) have revealed severe learning deficits^53^, decreased hippocampal volume and long-term potentiation critical for memory consolidation^33,54^ and impaired extinction learning^55^. Taken together, these studies suggest that neurobiological processes critical for memory function are modulated by BDNF, and that these processes can be disrupted as there is less efficient transcription, folding, and transport for the resultant BDNF protein in those with the Met allele^39^.

Several human studies support this influence of the BDNF Met allele on memory. Neuroimaging studies have shown that the hippocampus is integrally involved in memory formation and consolidation^33,56^ and some studies have shown that healthy human Met allele carriers have: smaller hippocampal volumes than Val/Val carriers^57–59^; reduced hippocampal activity during memory encoding and retrieval^38,60^ and lower levels of hippocampal *N*-acetylaspartate, a marker of neuronal integrity^37^. Lower grey matter volumes in the PFC in healthy Met carriers compared to Val/Val carriers have also been reported^61,62^ along with volumetric reductions in the amygdala which is functionally associated with the processing of fear and emotion^63,64^.

There have been few previous studies investigating the association between the Val66Met polymorphism and recognition memory. Two of these found that Met carriers had significantly lower recognition than Val/Val carriers^65,66^ while another found Met allele carriers had reduced hippocampal activity compared to Val/Val carriers in addition to lower recognition memory^38^. Less than a handful of studies have examined BDNF Val66Met in recognition memory using emotional stimuli. Of these, some found no genotypic differences in recognition memory tasks^59,67^, whereas one study found better recognition memory for positive and negative but not neutral stimuli in Met but not Val carriers^68^.

None of these studies have examined emotional recognition memory and BDNF in PTSD populations. Hori et al.^69^ is the only study to date that has examined the association of BDNF Val66Met SNP with emotional recognition memory in PTSD compared to healthy controls. They used a recognition memory task to calculate a measure of negative memory bias by subtracting the number of neutral recognition hits from negative hits. Results revealed that only PTSD patients with the Met allele had significantly greater negative memory bias than controls and that negative memory bias increased significantly with increasing numbers of Met alleles, suggesting that the relationship between PTSD and negative memory bias can be moderated by the Val66Met polymorphism. No studies to our knowledge have also modelled trauma exposure or compared PTSD and healthy controls with TE populations. A meta-analysis which examined BDNF Val66Met as a risk factor for PTSD reported that the extent of previous trauma exposure may be an important confound that could account for inconsistencies in findings and is critical to control in future studies^46^. Further, a recent BDNF stress-sensitivity model proposed that the timing, chronicity and intensity of stress/trauma exposure may interact with Val66Met to affect memory and neurobiological functioning^70^. This highlights the importance of including TE groups in BDNF research, and in controlling for the timing (child/adult) and extent (cumulative) of previous trauma exposure.

As only one study has examined the relationship between BDNF Val66Met SNP, PTSD and emotional memory, the current study aimed to further explore the relationship between the BDNF Val66met polymorphism and emotional recognition memory in PTSD. This study extends the previous research^69^ by including a measure of sensitivity (d-prime) as well as a measure of negative memory bias, including a TE group, and examining the effects of sex and the timing/extent of previous trauma exposure while controlling for variables such as ethnicity and BMI. It is hypothesized that BDNF Val66Met and PTSD will interact to influence emotional memory. Given the sparseness of the emotional recognition/BDNF/PTSD literature and the inconsistency of findings, this hypothesis is non directional.

## Results

### Demographic and clinical data

Table 1 outlines demographic and clinical data for the sample. There were no significant differences between the number of males and females in each group, however there were significant differences between groups in age, AUDIT (alcohol use), DASS anxiety, stress and depression scales, PCL scores and BMI and ethnicity. Games–Howell post-hoc tests revealed that Control participants were significantly younger and had lower BMI than both the TE group and the PTSD group, with no significant differences between the TE and PTSD groups in age (*p* = 0.95) and BMI (*p* = 0.40).

**Table 1:** Mean scores (standard deviations), significance and effect sizes of demographic and clinical measures for participants in the PTSD, TE and Control Groups.

Measure	Control (*n* = 85)	TE (*n* = 105)	PTSD (*n* = 44)	Total (*n* = 234)	Test statistic	*p*	Effect size ɳ_p_^2^
Age	21.88 (4.61)	27.52 (10.64)	28.11 (10.41)	25.59 (9.28)	*F* = 11.67	< 0.001	0.09
DASS Depression	2.31 (2.68)	2.99 (3.21)	8.34 (4.91)	3.75 (4.08)	*F* = 49.57	< 0.001	0.28
DASS Anxiety	2.18 (2.93)	2.61 (2.61)	8.07 (4.33)	3.48 (3.81)	*F* = 59.41	< 0.001	0.36
DASS Stress	3.47 (3.22)	5.06 (3.55)	11.66 (4.69)	5.72 (4.70)	*F* = 75.80	< 0.001	0.40
PCL	6.45 (8.01)	11.12 (8.77)	41.49 (14.33)	15.28 (16.29)	*F* = 156.6	< 0.001	0.55
AUDIT	4.25 (4.14)	5.11 (4.53)	8.21 (5.98)	5.37 (4.89)	*F* = 10.38	< 0.001	0.08
BMI	23.06 (3.61)	24.68 (5.12)	26.00 (5.80)	24.34 (4.88)	*F* = 5.96	< 0.001	0.05
Sex	48F, 37M	66F, 39M	30F, 14M	144F, 90M	χ^2^ = 0.40	0.67	
Meds	2 yes/83 no	7 yes/98 no	8 yes/36 no	17 yes/217 no	χ^2^ = 9.78	0.01	

*n* number of participants in group, *DASS* depression anxiety and stress scale; *PCL* PTSD Checklist, *AUDIT* alcohol use disorder identification test, *BMI* Body Mass Index; refer to Supplementary Information for medication type/distribution.

As expected, the PTSD group had significantly higher scores compared to the control and TE groups for PCL, AUDIT, and DASS depression, anxiety, and stress measures (all *p* < 0.001). There were no significant differences between TE and control groups for depression (*p* = 0.25), anxiety (*p* = 0.54) and AUDIT (*p* = 0.37) scores. However, the TE group had significantly higher scores than controls for PCL (*p* < 0.001) and Stress (*p* = 0.004). As depression, anxiety and stress were highly correlated, stress along with AUDIT scores, age and BMI were included in subsequent analyses as covariates to account for the significant differences in these variables across groups. Full post-hoc analyses for demographic and clinical measures are displayed in Supplementary Information (Table 1).

### BDNF allele frequency distribution and demographic results

Table 2 shows the frequency distribution of sex and BDNF genotype across groups. The Met allele carriers (Val/Met and Met/Met genotypes) were grouped together (into Val/Met) since the rarity of the Met/Met genotype in Caucasian populations can inhibit meaningful analysis^71^. The genotype frequencies of the 234 participants were 59% Val/Val (*n* = 139) and 41% Val/Met (*n* = 95). Genotype frequency did not differ across the groups (χ^2^ = 2.41, *p* = 0.30) and a Chi-square goodness of fit analysis revealed distribution of the BDNF genotype was in Hardy–Weinberg equilibrium (χ^2^ = 1.91, *p* = 0.12).

**Table 2:** Sex and BDNF genotype frequency distribution across groups.

	Genotype	Control	TE	PTSD	Total
Female	Val/Val	23	38	22	83
	Val/Met	25	28	8	61
	Total	48	66	30	144
Male	Val/Val	23	25	8	56
	Val/Met	14	14	6	34
	Total	37	39	14	90
Total		85	105	44	234

One-way ANOVAs were calculated to test for differences between genotypes across groups on demographic and clinical variables to identify any variables to be controlled in subsequent analyses. As expected from previous research^39^, these revealed significant differences between the genotype groups for BMI [*F* (1, 232) = 22.74, *p* = 0.001], AUDIT scores [*F* (1, 234) = 91.65, *p* = 0.05], and age [*F* (1, 237) = 5.14, *p* = 0.02]. Chi-square tests of independence for ethnicity × group were not significant for Val/Val [χ^2^ (4, *N* = 139) = 4.38, *p* = 0.36], but were significant for Val/Met participants [χ^2^ (2, *N* = 95) = 13.28 *p* = 0.001]. The proportion of participants from an Asian background relative to Caucasian background was greater in healthy controls, but opposite in the PTSD group who had a greater proportion of Caucasian compared to Asian participants (see Table 3). No other measures were significant (all *p* > 0.22). All models included ethnicity, BMI, age, AUDIT scores and stress as co-variates to account for the significant differences between genotypes for these variables.

**Table 3:** BDNF genotype and ethnicity frequency distribution across groups.

Genotype	Group	Ethnicity			Total
		Caucasian	Asian	Black	
Val/Val	Control	35 (76.1%)*	10 (21.7%)*	1 (2.2%)*	46 (100%)*
	TE	52 (82.5%)*	9 (14.3%)*	2 (3.2%)*	63 (100%)*
	PTSD	28 (93.3%)*	2 (6.7%)*	0	30 (100%)*
	Total	115 (82.7%)*	21 (15.1%)*	3 (2.2%)*	139 (100%)*
Val/Met	Control	12 (30.8%)*	27 (69.2%)*	0	39 (100%)*
	TE	23 (54.8%)*	19 (45.2%)*	0	42 (100%)*
	PTSD	12 (85.7%)*	2 (14.3%)*	0	14 (100%)*
	Total	47 (49.5%)*	37 (50.5%)*	0	95 (100%)*
Total	Control	47 (55.3%)*	37 (43.5%)*	1 (1.2%)*	85 (100%)*
	TE	75 (71.4%)*	28 (26.7%)*	2 (1.9%)*	104 (100%)*
	PTSD	40 (90.9%)*	4 (9.1%)*	0 (0.0%)*	44 (100%)*
	Total	162 (69.2%)*	69 (29.5%)*	3 (1.3%)*	234 (100%)*

*% within group.

### Recognition memory

#### d-prime measure

##### d-prime scores across valence

**Figure 1:**
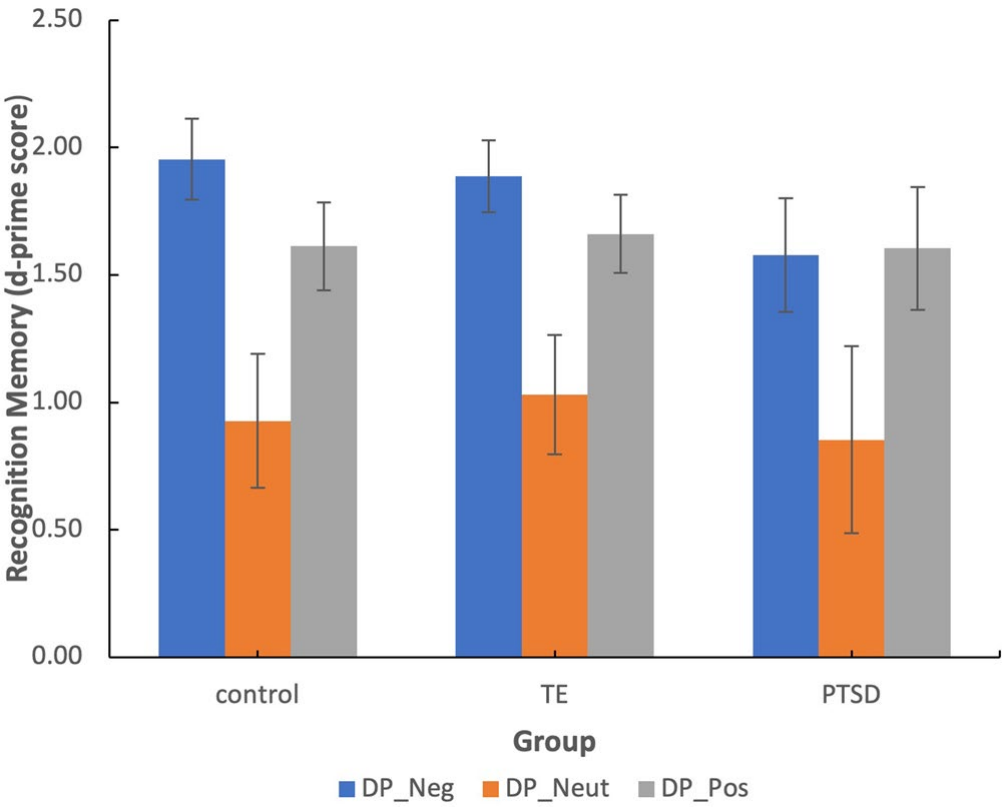
Mean d-prime scores for total recognition memory across the PTSD, TE and control groups. Error bars display: 95% confidence intervals.

A GLiM mixed linear model was used to compare recognition memory sensitivity (d-prime scores) for positive, negative and neutral images with the between subject factor of group and within subject factor of valence. Figure 1 displays the mean d-prime scores for positive, negative and neutral recognition memory in each group.

No significant main effect of group was found [*F* (2,741) = 1.71, *p* = 0.18], however there was a significant main effect of valence [*F* (2,741) = 37.36, *p* < 0.001] such that negative images were recognised significantly more than positive (*p* = 0.002, 95% CI [0.03, 0.33]) and neutral images (*p* < 0.001, 95% CI [0.63, 1.11]), and positive images were recognised significantly more than neutral images (*p* < 0.001, 95% CI [0.46, 0.92]). There was no significant group by valence interaction (*p* = 0.45).

##### Negative d-prime

Given that PTSD is characterised by predominant dysregulation in negative emotional memories and previous emotional recognition memory studies explored negative memory specifically^69^, to further explore negative d-prime results, an initial GLiM analysis was conducted to explore best model fit. The best fitting model based on the lowest AIC and BIC included predictors of group, BDNF genotype and sex with additional covariates of ethnicity and BMI. Results of this model showed a significant main effect of group [χ^2^ (2,* N* = 234) = 11.09, *p* = 0.004] and genotype [χ^2^ (1, *N* = 234) = 8.82, *p* = 0.003], but no significant main effect of sex [χ^2^ (1, *N* = 234) = 0.34, *p* = 0.56]. There was a group × genotype interaction effect [χ^2^ (2, *N* = 234) = 6.67, *p* = 0.036] however no further interactions were significant (all *p* > 0.25). There was no change to any of the predictor outcomes when stress, age and AUDIT scores were added as co-variates (all *p* > 0.05).

**Figure 2:**
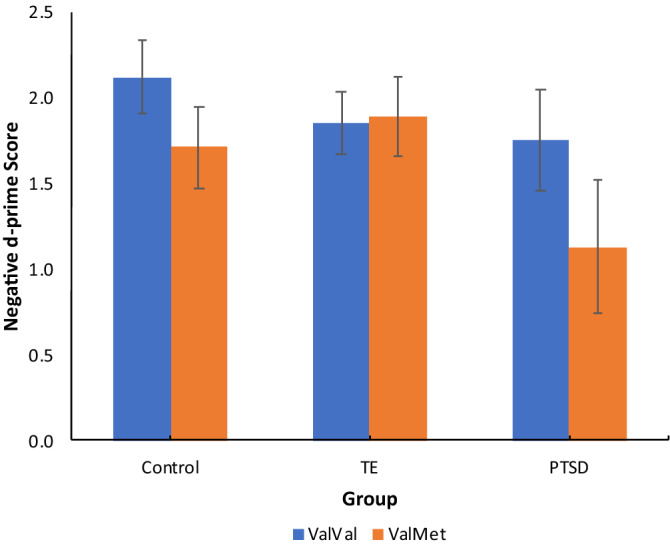
Mean negative d-prime scores for BDNF genotype across the PTSD, TE and control groups. Error bars: 95% confidence intervals.

Figure 2 displays the mean negative d-prime scores for each BDNF genotype across groups. The group × genotype interaction was analysed with post-hoc simple interaction effect tests. These revealed the significant group × genotype interaction was driven by the lack of difference between Val/Val and Val/Met in the TE group. This was supported by the significant group x genotype interactions between Control and TE [χ^2^ (1, *N* = 190) = 3.88, *p* = 0.05], PTSD and TE [χ^2^ (1, *N* = 149) = 4.83, *p* = 0.03], and the non-significant interaction effect for the PTSD and Control groups [χ^2^ (1, *N* = 129) = 0.57, *p* = 0.45]. There was a significant main effect of group [χ^2^ (2, *N* = 129) = 11.05, *p* = 0.001] with PTSD d-prime scores (*M* = 1.44, *SD* = 0.72) significantly lower than both Controls (*M* = 0.1.92, *SD* = 0.72) and TE groups (*M* = 0.1.86 *SD* = 0.83) and a significant genotype main effect with Val/Met d-prime scores (*M* = 1.42, *SD* = 0.76) significantly lower than the Val/Val genotype (*M* = 1.94, *SD* = 0.74).

#### Positive and neutral d-prime

The analyses applied to negative d-prime were repeated for d-prime scores for positive and neutral recognition memory. There were no significant effects for any of these analyses (all *p* > 0.77).

#### Sub-analysis: Timing of trauma exposure and extent of cumulative trauma

For the groups that had been previously exposed to trauma (TE and PTSD) in our analysis, the influence of timing of trauma exposure and cumulative trauma exposure were examined in separate models. The impact of timing of trauma on negative d-prime was explored by adding child versus adult trauma as an additional factor to the preceding GLiM model. The significant effects of group [χ^2^ (1, *N* = 145) = 9.11, *p* = 0.003], genotype [χ^2^ (1, *N* = 145) = 4.25, *p* = 0.04] and group × genotype interaction [χ^2^ (1, *N* = 145) = 8.73, *p* = 0.003] were found in the same direction as the original model. In addition, there was a significant group × child/adult trauma [χ^2^ (1, *N* = 145) = 3.87, *p* = 0.05] and group × sex × child/adult trauma interaction effect [χ^2^ (1, *N* = 145) = 4.24, *p* = 0.04] but no other significant main or interaction effects (all *p* > 0.07). The interaction revealed that negative d-prime scores for those who first experienced trauma as an adult were significantly higher in the TE than PTSD groups, however there were no significant differences in d-prime scores between TE and PTSD for participants who first experienced trauma as a child. From the three-way interaction, it appeared female PTSD participants who first experienced trauma as an adult were most affected with significantly lower negative d-prime scores (see Supplementary Information for further details). There were no significant interactions between BDNF Genotype and timing of trauma.

To control for the impact of cumulative trauma exposure, the number of traumas experienced was then added as a covariate (in addition to ethnicity and BMI) to the initial negative d-prime GLiM analysis in the TE and PTSD groups. Cumulative effect of trauma did not significantly interact with any variable in the model resulting in the same significant effects as the original model of group [χ^2^ (1, *N* = 149) = 8.29, *p* = 0.004], genotype [χ^2^ (1, *N* = 149) = 3.87, *p* = 0.05] and group × genotype interaction [χ^2^ (1, *N* = 149) = 4.83, *p* = 0.03].

In considering childhood trauma, another way of modelling the impact of cortisol and glucocorticoid sensitivity which is relevant for BDNF is to examine FKBP5. As a sub-analysis, FKBP5 was entered as a factor however there were no significant main or interaction effects with BDNF in any of these analyses.

#### Additional negative memory measures

**Figure 3:**
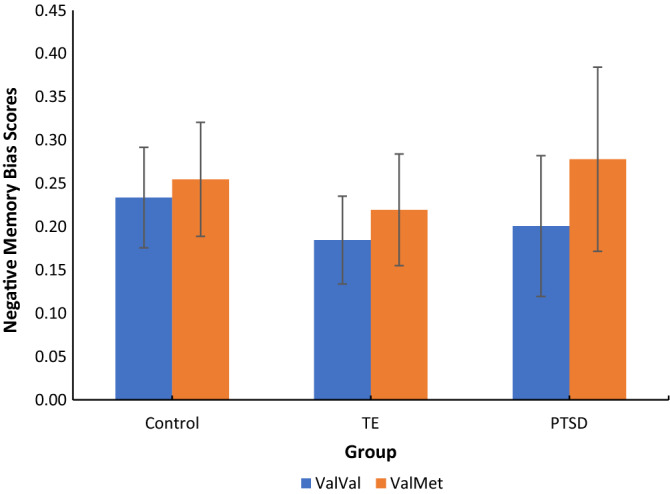
Mean negative bias scores for BDNF genotype across the PTSD, TE and control groups. Error bars: 95% confidence intervals.

Negative bias, hit, and false alarm rates were also analysed using GLiM models (see Supplementary Information for hit and false alarm results). As per negative d-prime scores, the best model fit for all measures included predictors of group, BDNF and sex, with ethnicity and BMI included as covariates. There was no change to any of the predictor outcomes when stress, age and AUDIT scores were added as covariates (all *p* > 0.05). Figure 3 displays the mean negative bias scores for BDNF genotype across groups. No significant group (*p* = 0.35), genotype (*p* = 0.12), group × genotype (*p* = 0.79) or other main/interaction effects were found. The addition of child/adult and cumulative trauma to the model did not have any significant effects.

## Discussion

This study investigated the interactive effects of BDNF Val66Met genotype and PTSD symptomatology in an emotional recognition memory task. Key findings revealed that the PTSD group had impaired negative recognition memory compared to Control and TE groups, with no group differences in positive and neutral recognition. Negative recognition memory was impaired in participants with the BDNF Val/Met genotype compared to Val/Val, and there was an interaction between group and genotype. This interaction revealed that in both PTSD and healthy controls, participants with the Met allele had lower recognition scores than those with the Val allele. However the TE group showed no significant genotypic differences in recognition memory. This Val66Met genotype effect was not influenced by timing or number of traumas experienced, and remained when controlling for sex, ethnicity, BMI, age and other covariates. The timing of trauma interacted with group independently of BDNF genotype, revealing that if trauma was first experienced as an adult, patients with PTSD had poorer negative recognition memory than TE participants, with no group differences in memory for those with childhood trauma exposure.

Our study findings are consistent with several previous emotional recognition memory studies showing no differences between groups in positive and neutral recognition^19,20,22–24^. Results of previous studies examining negative recognition memory have been inconsistent, with some finding better recognition memory in PTSD groups compared to controls^26,27^ or no differences between groups^29,30^. This may have been due to methodological variation in measures and tasks between the studies. Only one other study examining negative recognition memory in PTSD also found decreased net performance in recognition memory in PTSD compared to controls^28^, however they used words instead of images as stimuli and did not include a TE comparison group.

Our study also found a main effect of BDNF Val66Met genotype where participants with the Val/Met genotype had decreased negative recognition memory compared to those with the Val/Val genotype. This accords with previous studies^38,65,66^ which utilised non-emotional stimuli. However our findings of decreased recognition memory for negative stimuli in Met carriers but not Val homozygotes are divergent from the few previous recognition memory studies to use emotional stimuli. Two prior studies found no genotypic differences in emotional recognition memory^59,67^ however these studies were significantly limited by power. One of these studies^67^ had only *N* = 47 participants, and although the other study^59^ had *N* = 157, only *n* = 31 of these were in the control group. A subsequent study found better recognition memory for positive and negative but not neutral stimuli in Met compared to Val carriers^68^, however again power was significantly limited (*N* = 36). These previous studies did not include comparisons with PTSD or TE groups or control for variables like ethnicity, BMI or trauma exposure. This highlights the need for further research to better elucidate findings and for future research to control for these factors along with variables such as age, alcohol use and other relevant demographic factors.

Hori et al.^69^ is the only other study to our knowledge to examine the association of BDNF Val/Met with PTSD using an emotional memory paradigm. Using a measure of negative memory bias (measured by subtracting total neutral hits from negative hits), they found that negative memory bias increased with increasing numbers of Met allele only in the PTSD group, but not in healthy controls. We also calculated a negative memory bias measure and found a similar pattern, however our results did not reach significance and displayed small effect sizes. The same method of calculation for negative bias was used in both studies but due to different recognition memory tasks (Japanese Kanji vs images) and different time periods between encoding and recognition phases in the two studies, negative memory bias was not an identical measure. The ethnicity of participants between studies was also very different which may have influenced outcomes. Additionally, Hori et al. did not include a TE group, examine the timing or cumulative effects of trauma, or use a measure of sensitivity such as d-prime for a more nuanced comparison.

As predicted, the current study found a significant interaction between BDNF genotype and group. Although there was a significant Met recognition memory impairment in the PTSD group, this was not specific to PTSD but also featured in healthy controls. As illustrated in Fig. 2, what appears to be driving this interaction effect is the very different pattern displayed in the TE group, where the TE group appear to be protected from the impact of the BDNF Val66Met genotype on emotional recognition memory. To our knowledge, no previous studies have examined the impact of BDNF Val66Met on emotional memory comparing PTSD with TE populations. However, two previous studies have examined the influence of the BDNF Val66Met genotype in PTSD vs TE groups on general cognition^72,73^ and found cognitive deficits in participants with the BDNF Met allele in the PTSD, but not the TE groups. This lack of cognitive deficit in TE participants with the Met allele relative to patients with PTSD aligns with our current findings.

The absence of a BDNF Val66Met genotypic recognition memory effect in the TE group in our study is an unexpected finding and needs replication but appears to be robust to potential confounds. These results were not due to uneven genotype distribution as there were no significant differences in BDNF distribution across the three groups. The possibility these results may have been influenced by uneven ethnicity distribution across the three groups (i.e. a greater proportion of people with Asian backgrounds in the control group and a greater proportion of Caucasian participants in the PTSD group) was controlled for by adding ethnicity as a covariate which did not change our findings. Further, when analyses were run using only Caucasian participants, results followed a very similar pattern (see Supplementary Information).

As can be seen from the clinical data in Table 1, although the TE group experienced a Criterion A trauma, they were a psychologically resilient group, reporting PTSD symptoms only slightly above that of non TE controls. A recent theory based on rodent research^70^ proposes a stress-sensitivity hypothesis which suggests that the BDNF Val66Met polymorphism impact on neural function and behaviour relevant for memory is influenced by the chronicity and intensity of stress/trauma exposure via glucocorticoid interactions with BDNF, potentially via DNA methylation or other epigenetic mechanisms^62,74,75^. Therefore there may be a potential neuroprotective mechanism in this TE population which minimizes the impact of the BDNF Val66Met genotype and promotes resilience to stress-related disorders. One possibility may be that the TE group is characterized by less DNA methylation of BDNF which could make them less influenced by the Met genotype and more resilient to developing PTSD. Alternatively, the glucocorticoid signalling in the TE group may adaptively protect against the impact of BDNF Val66Met, promoting resilience to PTSD. These possibilities need exploration in future research examining BDNF Val66Met genotype, glucocorticoid function and epigenetics. As the authors^70^ note, a critical gap in the literature is understanding what role the impact of trauma exposure has on BDNF function in relation to PTSD.

Allelic frequency of the Val66Met SNP differs greatly between ethnicities and sub-population groups with the Met allele more common in Asian compared to Caucasian populations and virtually absent in African and native American populations^48,76^. One of the strengths of our study is that ethnicity was included as a covariate to account for this influence and the fact most of our participants were Caucasian, however the cognitive and behavioural effects associated with the Met genotype have also been observed to be more robust in Caucasians^39,48^. Further, due to the rarity of the Met/Met genotype, many studies using Caucasian samples combine Met/Met and Val/Met participants for comparison against Val/Val participants^74^ as in the current study. This was necessary due to limited numbers but may have inadvertently reduced the strength of the effect. Thus, genotype compared to allelotype effects may not be revealed, particularly among Caucasian samples^39^. As this effect has been well documented in animal studies^34,54^, future studies should recruit large enough sample sizes to examine triallelic effects.

There were some limitations in the current study. This research is a single candidate genotype study, the advantage being that it is hypothesis driven enabling investigation at a mechanistic level. However, for greater robustness, ideally the study should also have been informed by GWAS and polygenic risk scores. Our sample size prevented these types of analyses, but future research should investigate this using larger data sets. A lack of power due to the smaller number of PTSD participants in comparison with other groups may have also influenced results. A larger sample size could improve power to better test sex specificity in BDNF expression, which is known to have sex specific effects and be influenced by oestrogen^39,77^ and thus should be an important issue for future consideration. Due to the fact our participants were a non-treatment seeking sample collected in a university setting rather than a treatment clinic, a structured diagnostic interview for PTSD was not conducted which is a limitation of the study. While the PCL-5 is a validated measure of symptom severity enabling a probable diagnosis, future research should employ diagnostic interviews where possible.

In conclusion, this study is the first to our knowledge to compare the influence of BDNF Val66Met on emotional recognition memory in PTSD, TE and healthy controls. Results replicated previous literature in finding lower negative emotional recognition memory in PTSD and Val/Met populations. The study also extended previous literature in comparing the three groups, taking into account the timing and number of traumas experienced and in using a measure of sensitivity (d-prime, the most widely used recognition memory measure), which revealed the BDNF Met effect occurred in both PTSD and healthy controls, but not in the TE group. This result has not been found in previous literature and could imply that people who have been exposed to trauma but do not have PTSD may have some protection from the BDNF Met effect. However more research is needed to replicate these results and to explore the relevant epigenetic and neural processes involved. A minor limitation of the current study was the lack of a general measure of cognitive performance to test whether the Val66Met effect was a specific memory processing deficit as opposed to a general effect on cognitive performance, as BDNF Val66Met is known to affect working memory and processing speed^31^. Although studies concerning this have shown no consistent effect to date^32^, future studies could add more certainty by including such measurements.

## Methods

### Participants

The study was completed as part of a larger emotional memory study where delayed recall and intrusive memories were also collected in a sample of 307 participants. Data reported in this study is from a recognition memory task completed by a subset of participants (*N* = 234) from this larger study. The study was conducted on two separate sites at the Universities of Tasmania and Melbourne with 144 females and 90 males (age *M* = 25.6) completing the recognition study. Participants included students from both universities and members of the public recruited through advertisements posted throughout the community, universities, and in various private psychology clinics. Participants either received course credit or $100 for participating. The study was approved by both the Tasmanian and Victorian Medical Human Research Ethics Committees. All experiments were performed in accordance with relevant guidelines and regulations.

The control group consisted of 85 participants (48 female, 37 male, age *M* = 21.9, *SD* = 4.6) with no exposure to a Criterion A traumatic event as outlined by the Traumatic Events Questionnaire (TEQ)^78^ and minimal symptoms on the PTSD Checklist (PCL 5)^79^. One hundred five participants (66 female, 39 male, age *M* = 27.5, *SD* = 10.6) were classified as TE having experienced a Criterion A traumatic event but did not meet DSM-5 diagnostic criteria for PTSD. Forty-four participants (30 female, 14 male, age *M* = 28.1, *SD* = 10.4) were classified as PTSD having experienced a Criterion A traumatic event and reported symptoms consistent with DSM-IV-TR or DSM-5 diagnostic criteria for PTSD on the PCL^79^.

The PTSD group were a civilian sample with a range of traumas including sexual or interpersonal assault, combat/war zone experience, natural disasters and motor vehicle accidents (MVA). Exclusions included anyone reporting neurological damage, head injury, substance or alcohol dependence, psychosis, suicidality, serious medical illness and women who were pregnant or breast feeding. All participants were under the age of 65 to control for potential memory confounds. Medications recorded included anti-depressants, anxiolytics, benzodiazepines and mood stabilisers (see Supplementary Information for breakdown across groups). Females were also asked to record whether or not they used oral contraceptives however as a large number of participants did not respond to this question, there was not sufficient numbers to enable meaningful analysis. Control group participants reported no psychiatric history or psychoactive medication use however two control participants reported using amitriptyline for rheumatoid issues (arthritis, fibromyalgia) rather than as an anti-depressant).

### Materials and measures

#### International affective picture system images (IAPS)^80^

IAPS images were selected according to their valence (ranging from unpleasant or negative to pleasant or positive rated 1–9) and arousal (calm to exciting rated 1–9). Twenty emotionally negative images (mean valence: 2.30, arousal: 6.18), 20 neutral images (mean valence: 4.99, arousal: 2.75) and 20 positive images (mean valence: 7.49, arousal: 4.42) were selected using normative data and stimuli from the IAPS and displayed on a computer screen.

#### Depression, anxiety and stress scale (DASS)^81^

The DASS is a 21 item self-report questionnaire assessing the severity of current depressed, anxious and stress mood states. Each item is rated on a four-point Likert Scale of severity or frequency of the participants’ experiences over the last week. Scores range from 0 (“did not apply at all”) to 3 (“applies very much, or most of the time”). Reliability of the scales in terms of Cronbach’s Alpha rate the anxiety scale at 0.84, stress at 0.90 and depression at 0.91 in the normative sample. This scale was used to index the level of depressed, anxious and stressed mood during the week of testing.

#### PCL^79^

The PCL was used to screen for PTSD symptomatology. The PCL-5 was used at the Melbourne testing site while an earlier version (PCL-IV) was used in Hobart. The PCL-5 is a standardized 20-item self-report measure of PTSD symptomatology corresponding to the DSM-5 symptom criteria for PTSD. Respondents indicate the extent they were affected by each symptom in the past month using five-point Likert scales with scores ranging from 1 “Not at all” to 5 “Extremely”. The PCL provides an ordinal measure of the severity of PTSD symptoms and suggests a cut-off of 31–33 for probable PTSD. A symptom is scored as “moderate” is considered a symptom endorsement, and diagnostic status can be obtained by examining a pattern of endorsement of the 20 PTSD symptoms as per DSM-5 (APA, 2013). PCL-5 scores range from 0 to 80. The PCL-IV is a 17 item self-report measure that mapped onto DSM-IV-TR PTSD symptom criteria. Scores range from 17 to 85.

#### TEQ^78^

The TEQ (Cronbach’s α = 0.91) is an 11-item dichotomous (yes/no) scale reflecting if respondents have experienced a category A trauma (DSM-5, APA 2013) providing an index of the nature of the traumatic event experienced. The TEQ was used to screen for trauma exposure.

#### Alcohol use identification test (AUDIT)^82^

The AUDIT is a 10-item questionnaire covering the domains of alcohol consumption, drinking behaviour and alcohol related problems. Responses are scored on a scale of 0–4 with a maximum score of 40. Harmful is determined by a score of 8 or more and alcohol dependence a score of 16 or above.

### Procedure

**Figure 4:**
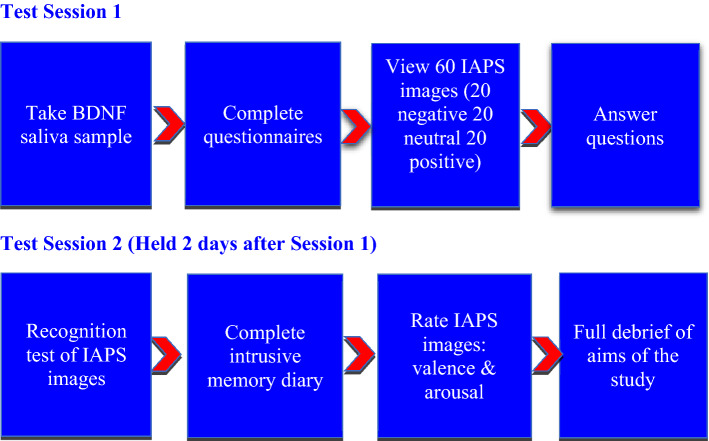
Experimental timeline.

Participants individually completed two testing sessions 2 days apart. In the first session, participants gave informed consent and were habituated to the test environment for 10 min. Questionnaires were administered and a saliva sample taken for DNA extraction for genotyping BDNF Val66Met. Participants then viewed a series of images on a computer screen. Sixty IAPS images were presented, 20 negative (rated as highly unpleasant and arousing), 20 neutral and 20 positive (pleasant but not arousing). Images were shown for 6s each in block format (20 positive, 20 negative, 20 neutral) in randomised order across participants. Participants were then informed they were to return in two days when they would undertake similar procedures including rating each IAPS image on valence and arousal following standardised procedures^80^. Participants were not informed they would complete a memory test at that time to prevent priming or image rehearsal. Figure 4 outlines a timeline of the procedure of the study.

At the second testing session, in a surprise memory task, participants were presented with the same 60 images (20 negative, 20 neutral and 20 positive) as in the encoding task, in addition to 60 new images of similar aversiveness and valence which had not been presented previously. Images were shown for 6s each in block format of positive, negative and neutral (40 images in each block: 20 old and 20 new), in randomised order across participants. In accordance with van Ast et al.^83^, participants were told the images might be old (presented in the encoding task) or new (not previously presented) and were asked to rate each image on a 6-point Likert scale from ‘definitely old’ to ‘definitely new’. Any rating 3 or under was categorised as `old’ with ratings 4 and above a ‘new’ response. A full debrief concerning the aims of the research was given on completion.

### Design and analysis

#### Salivary genomic analysis

Participants provided a 2 mL saliva sample using Oragene DNA self-collection kits (DNA Genotek Inc, 2012). Purification and extraction of DNA from saliva samples was performed following standard methods provided by DNA Genotek Inc. and carried out in the pathology labs of the UTAS School of Medicine. The BDNF Val66Met polymorphism was identified using an established polymerase chain reaction (PCR) method^84^. PCR amplifications were conducted using a 10µL reaction volume containing approximately 50 mg of genomic DNA. PCR amplicons were resolved on a 2% agarose gel. Genotyping was repeated to ensure accuracy, with the proportion of concordance > 99%.

#### Statistical analysis

Analyses were performed on SPSS 27 for Windows. Clinical and demographic data were analysed with univariate analyses of variance (ANOVA) to test for group differences. Greenhouse–Geisser (GG) corrections were used where significant sphericity appeared in the data. Sex, medication, ethnicity and genotype distribution were analysed using 2 × 3 Chi-square tests of independence. For PCL scores, due to use of PCL-IV and PCL-5 at different testing sites, a PCL ‘Crosswalk’ was used to convert PCL-IV to PCL-5 symptom severity scores as per Moshier et al.^85^. A Chi-square goodness of fit test was used to compare observed genotype frequencies with the expected genotype frequencies for an Australian population. This was to determine if the sample was significantly different from Hardy–Weinberg equilibrium to ensure that the distribution of genotype alleles matched that of the general population^86^.

For memory data, d-prime scores were used as a measure of sensitivity, in which a single value reflects accuracy in both correctly identifying old target images and correctly rejecting novel images. This was calculated according to signal detection theory^87^ for each valence category by subtracting the z-scored false alarm from the z-scored hit scores, with hit and miss scores dichotomised from the 1 to 6 Likert data. The number of negative images previously viewed and correctly recognised as old constituted negative hit rates. The number of images falsely recognised as old constituted false alarm rates. Negative memory bias was calculated by subtracting hit rates of neutral items from those of negative items.

A mixed linear model was used to compare differences between d-prime valence measures (positive, neutral and negative) across the three groups and Generalised Linear Model (GLiM) logit link individually compared each type of negative recognition memory (d-prime, hit, false alarm and negative bias scores) as response variables against predictor variables of group, genotype and sex with ethnicity and BMI as covariates. Sex was entered as a specific predictor in the model as research demonstrates there is sex specificity where the BDNF effect is stronger in females than males. BMI and ethnicity are known confounds which can influence BDNF and cause high heterogeneity and were included as covariates in the models to remove these confounding effects.

Period of trauma (child/adult) was then subsequently added as a factor and then in a separate GliM, number of traumas experienced was added as a covariate (see Supplementary Information for table outlining each GLiM and predictor/covariate combinations analysed) Sequential Sidak post-hoc analyses were used where appropriate. Alpha values of *p* < 0.05 were set for significance testing for all statistical analyses.

## Supplementary Information

Supplementary Information.

## Data Availability

The datasets analysed for this study are available from the corresponding author, E.L.N., upon reasonable request.
