# Supplementary material for: The influence of the BDNF Val66Met genotype on emotional recognition memory in post-traumatic stress disorder
Source: Sci Rep. 2023 Mar 28;13:5033. doi: 10.1038/s41598-023-30787-6 (PMC10050310; doi:10.1038/s41598-023-30787-6)
Supplement: Supplementary file 1 — Supplementary Information. [file 41598_2023_30787_MOESM1_ESM.docx]

**Supplementary Information**

The influence of the BDNF Val66Met genotype on emotional recognition memory in post-traumatic stress disorder

Emma Louise Nicholson^1*^, Michael Garry^2^, Luke J. Ney^2,3^, Chia-Ming K. Hsu^2^, Daniel V. Zuj^2,4^ and Kim L. Felmingham^1^

^1^Melbourne School of Psychological Sciences, University of Melbourne, Australia

^2^School of Psychological Sciences, University of Tasmania, Australia

^3^School of Psychology and Counselling, Faculty of Health, Queensland University of Technology, Australia

^4^Experimental Psychopathology Lab, Department of Psychology, Swansea University, UK

^*^Corresponding author: Emma Nicholson, Melbourne School of Psychological Sciences, Redmond Barry Building, University of Melbourne Parkville VIC 3010 Australia

### Email address: elnicholson@student.unimelb.edu.au

Phone number: +61405978831

Table 1

*Significant Sidak Pairwise Comparisons With p-values and 95% CIs Following Up Significant ANOVAs for Demographic Clinical Measures Across Groups*

|  |  |  | 95% Confidence Intervals | | |
| --- | --- | --- | --- | --- | --- |
| Measure | Groups compared | *p* value | | Lower bound | Upper bound |
| Age | Controls and TE | <.001 | 2.52 | | 8.76 |
|  | Controls and PTSD | .001 | 2.26 | | 10.20 |
| BMI | Controls and TE | *.*031 | .12 | | 3.12 |
|  | Controls and PTSD | .009 | .63 | | 5.24 |
| DASS |  |  |  | |  |
| - depression | PTSD and TE | < .001 | 3.42 | | 7.28 |
|  | PTSD and controls | < .001 | 4.12 | | 7.95 |
| - anxiety | PTSD and TE | < .001 | 3.77 | | 7.15 |
|  | PTSD and controls | < .001 | 4.15 | | 7.63 |
| - stress | PTSD and TE | < .001 | 4.74 | | 8.46 |
|  | PTSD and controls  TE and controls | < .001  .004 | 6.33  .42 | | 10.05  2.75 |
| PCL | PTSD and TE | < .001 | 24.50 | | 34.08 |
|  | PTSD and controls | < .001 | 31.62 | | 41.53 |
| AUDIT | PTSD and TE | < .001 | .67 | | 5.54 |
|  | PTSD and controls | .001 | 1.52 | | 6.40 |
|  |  |  |  | |  |

Table 2a.

*Mean Negative D-Prime Scores across Group, Sex and Genotype*

| Group | Genotype | Sex | Mean | *SE* | *n* |
| --- | --- | --- | --- | --- | --- |
| Control | Val/Val | Male | 2.2253 | .15161 | 23 |
|  |  | Female | 2.0236 | .15161 | 23 |
|  | Val/Met | Male | 1.6400 | .19432 | 14 |
|  |  | Female | 1.7910 | .14542 | 25 |
| TE | Val/Val | Male | 1.7174 | .14542 | 25 |
|  |  | Female | 1.9985 | .11795 | 38 |
|  | Val/Met | Male | 1.9436 | .19432 | 14 |
|  |  | Female | 1.8422 | .13741 | 28 |
| PTSD | Val/Val | Male | 1.6975 | .25706 | 8 |
|  |  | Female | 1.8102 | .15502 | 22 |
|  | Val/Met | Male | 1.0585 | .29683 | 6 |
|  |  | Female | 1.2064 | .25706 | 8 |

*Note:* SE= standard error, *n* =number of participants

Table 2b.

*Mean Negative D-Prime Scores across Groups*

| Group | Mean | Standard Error | *n* |
| --- | --- | --- | --- |
| Control | 1.9200 | .08096 | 85 |
| TE | 1.8754 | .07571 | 105 |
| PTSD | 1.4432 | .12357 | 44 |

Table 2c.

*Mean Negative D-Prime Scores across BDNF Genotype*

| Genotype | Mean | Standard Error | *n* |
| --- | --- | --- | --- |
| Val/Val | 1.9121 | .06895 | 139 |
| Val/Met | 1.5803 | .08656 | 95 |

Table 2d.

*Mean Negative D-Prime Scores across Sex*

| Sex | Mean | Standard Error | *n* |
| --- | --- | --- | --- |
| Male | 1.7137 | .08722 | 90 |
| Female | 1.7786 | .06812 | 144 |

Table 3a.

*Mean Negative D-Prime Scores across Groups with Child/Adult trauma*

| Group | Mean | Standard Error | *n* |
| --- | --- | --- | --- |
| TE | 1.8806 | .07795 | 101 |
| PTSD | 1.4941 | .09487 | 44 |

Table 3b.

*Mean Negative D-Prime Scores across BDNF Genotype with Child/Adult Trauma*

| Genotype | Mean | Standard Error | *n* |
| --- | --- | --- | --- |
| Val/Val | 1.7956 | .08498 | 92 |
| Val/Met | 1.5912 | .08662 | 53 |

Table 3c.

*Mean Negative D-Prime Scores across Sex with Child/Adult Trauma*

| Sex | Mean | Standard Error | *n* |
| --- | --- | --- | --- |
| Male | 1.6702 | .10682 | 52 |
| Female | 1.7265 | .06505 | 93 |

Table 3d.

*Mean Negative D-Prime Scores across Child/Adult Trauma*

| Genotype | Mean | Standard Error | *n* |
| --- | --- | --- | --- |
| Child (<18yrs) | 1.6069 | .08935 | 79 |
| Adult (18 and above) | 1.7265 | .08068 | 66 |

Table 3e

*Significant Main and Interaction Effects for Negative D-Prime Scores with Child/Adult Trauma Included as a Factor*

| Significant Effect | N | *df* | χ ^2^ | *p* |
| --- | --- | --- | --- | --- |
| Group | 145 | 1 | 9.11 | .00 |
| Genotype | 145 | 1 | 4.25 | .04 |
| Group x Genotype | 145 | 1 | 8.73 | .00 |
| Group x Child/Adult | 145 | 1 | 3.87 | .05 |
| Group x Child/Adult x Sex | 145 | 1 | 4.24 | .04 |

Table 4a.

*Mean Negative Hit Rate Scores across Group, Sex and Genotype*

| Group | Genotype | Sex | Mean | *SE* | *n* |
| --- | --- | --- | --- | --- | --- |
| Control | Val/Val | Male | .9000 | .02174 | 23 |
|  |  | Female | .8957 | .02360 | 23 |
|  | Val/Met | Male | .8536 | .02049 | 14 |
|  |  | Female | .8480 | .02735 | 25 |
| TE | Val/Val | Male | .8340 | .03865 | 25 |
|  |  | Female | .8697 | .01893 | 38 |
|  | Val/Met | Male | .8393 | .03202 | 14 |
|  |  | Female | .8107 | .02850 | 28 |
| PTSD | Val/Val | Male | .8250 | .04760 | 8 |
|  |  | Female | .8636 | .03086 | 22 |
|  | Val/Met | Male | .8167 | .04953 | 6 |
|  |  | Female | .7312 | .06246 | 8 |

Table 4b.

*Mean Negative Hit Rate Scores across Groups*

| Group | Mean | S.E. | *n* |
| --- | --- | --- | --- |
| Control | .8743 | .08096 | 85 |
| TE | .8384 | .07571 | 105 |
| PTSD | .8091 | .12357 | 44 |

Table 4c.

*Mean Negative Hit Rate Scores across BDNF Genotype*

| Genotype | Mean | SE | *n* |
| --- | --- | --- | --- |
| Val/Val | .8647 | .01302 | 139 |
| Val/Met | .8166 | .01612 | 95 |

Table 4d.

*Mean Negative Hit Rate Scores across Sex*

| Sex | Mean | SE | *n* |
| --- | --- | --- | --- |
| Male | .8448 | .01503 | 90 |
| Female | .8365 | .01427 | 144 |

Table 5a.

*Mean Negative False Alarm Scores across Group, Sex and Genotype*

| Group | Genotype | Sex | Mean | *SE* | *n* |
| --- | --- | --- | --- | --- | --- |
| Control | Val/Val | Male | .2283 | .03102 | 23 |
|  |  | Female | .2848 | .04491 | 23 |
|  | Val/Met | Male | .3214 | .04187 | 14 |
|  |  | Female | .2920 | .04115 | 25 |
| TE | Val/Val | Male | .3200 | .03394 | 25 |
|  |  | Female | .2513 | .02957 | 38 |
|  | Val/Met | Male | .2250 | .02846 | 14 |
|  |  | Female | .2196 | .02235 | 28 |
| PTSD | Val/Val | Male | .2750 | .03423 | 8 |
|  |  | Female | .3023 | .03317 | 22 |
|  | Val/Met | Male | .4833 | .06736 | 6 |
|  |  | Female | .3250 | .05796 | 8 |

Table 5b.

*Mean Negative False Alarm Scores across Groups*

| Group | Mean | S.E. | *n* |
| --- | --- | --- | --- |
| Control | .2816 | .02004 | 85 |
| TE | .2540 | .01444 | 105 |
| PTSD | .3464 | .02521 | 44 |

Table 5c.

*Mean Negative False Alarm Scores across BDNF Genotype*

| Genotype | Mean | SE | *n* |
| --- | --- | --- | --- |
| Val/Val | .2769 | .01422 | 139 |
| Val/Met | .3111 | .01875 | 95 |

Table 5d.

*Mean Negative False Alarm Scores across Sex*

| Sex | Mean | SE | *n* |
| --- | --- | --- | --- |
| Male | .8448 | .01503 | 90 |
| Female | .8365 | .01427 | 144 |

Table 6a.

*Mean Negative Bias Scores across Group, Sex and Genotype*

| Group | Genotype | Sex | Mean | *SE* | *n* |
| --- | --- | --- | --- | --- | --- |
| Control | Val/Val | Male | .2978 | .04637 | 23 |
|  |  | Female | .1696 | .03582 | 23 |
|  | Val/Met | Male | .2679 | .04274 | 14 |
|  |  | Female | .2420 | .04342 | 25 |
| TE | Val/Val | Male | .1720 | .04900 | 25 |
|  |  | Female | .1974 | .03511 | 38 |
|  | Val/Met | Male | .2750 | .04482 | 14 |
|  |  | Female | .1643 | .03588 | 28 |
| PTSD | Val/Val | Male | .2063 | .03897 | 8 |
|  |  | Female | .1955 | .03549 | 22 |
|  | Val/Met | Male | .3250 | .11472 | 6 |
|  |  | Female | .2313 | .06308 | 8 |

Table 6b.

*Mean Negative Bias Scores across Groups*

| Group | Mean | S.E. | *n* |
| --- | --- | --- | --- |
| Control | .2443 | .02113 | 85 |
| TE | .2022 | .02081 | 105 |
| PTSD | .2395 | .03528 | 44 |

Table 6c.

*Mean Negative Bias Scores across BDNF Genotype*

| Genotype | Mean | SE | *n* |
| --- | --- | --- | --- |
| Val/Val | .2064 | .01654 | 139 |
| Val/Met | .2509 | .02590 | 95 |

Table 6d.

*Mean Negative Bias Scores across Sex*

| Sex | Mean | SE | *n* |
| --- | --- | --- | --- |
| Male | .2573 | .02531 | 90 |
| Female | .2000 | .01742 | 144 |

Table 7.

*Number of Participants on Medication & Medication Type across Groups*

| Medication | Control | TE | PTSD (%) |
| --- | --- | --- | --- |
| No of participants on medication | 2 | 7 | 8 |
| Anti-depressants | 2 (100% - amitriptyline prescribed for pain not depression) | 6 (86%) | 7 (88%) |
| Anxiolytics | 0 | 1 (14%) | 3 (38%) |
| Mood stabilisers | 0 | 0 | 2 (25%) |
| Benzodiazepines | 0 | 0 | 3 (38%) |

*Note:* In the PTSD group, 4 participants noted taking multiple medication types – 4 participants used only anti-depressants; TE reported single type medications only

Table 8.

*Generalised Linear Model Variable Combinations Analysed*

| Model | Response Variable | Predictor Variables | Co-variates |
| --- | --- | --- | --- |
| Generalised Linear Model (GLiM) | Negative d’ | Group  Val66Met Genotype  Sex | Ethnicity  BMI |
| GLiM | Negative d’ | Group  Val66Met Genotype  Sex | Ethnicity  BMI  Stress |
| GLiM | Negative d’ | Group  Val66Met Genotype  Sex | Ethnicity  BMI  Age |
| GLiM | Negative d’ | Group  Val66Met Genotype  Sex | Ethnicity  BMI  AUDIT scores |
| GLiM | Negative d’ | Group (TE & PTSD only)  Val66Met Genotype  Sex  Child/Adult trauma | Ethnicity  BMI |
| GLiM | Negative d’ | Group (TE & PTSD only)  Val66Met Genotype  Sex | Ethnicity  BMI  Number of traumas experienced |
| GLiM | Negative Bias Score | Group  ValMet Genotype  Sex | Ethnicity  BMI |
| GLiM | Negative Bias Score | Group (TE & PTSD only)  ValMet Genotype  Sex  Child/Adult trauma | Ethnicity  BMI |
| GLiM | Negative Bias Score | Group (TE & PTSD only)  ValMet Genotype  Sex | Ethnicity  BMI  Number of traumas experienced |

*Note:* As per the initial models with negative d’ as the dependent variable (see above in red text), all subsequent models included separate analyses with stress, age, and audit scores added individually as covariates in addition to ethnicity, BMI and number of traumas experienced.

^a^ Separate GLiM models were also analysed using the above predictor combinations for hits and false alarms (see supplementary table for these results)

*Figure 1*. Mean false alarm scores for BDNF genotype across the PTSD, TE and control groups. There was a significant group [χ ^2^ (2, *N*=234) = 7.62, *p*=.02] and group x genotype [χ ^2^ (2, *N*=234) = 8.94, *p*=.01] but no other significant main or interaction effects (all *p* >.12). Follow up simple interaction effects indicated the TE group differences in genotype were the opposite to the differences in the control and PTSD groups with a significant group x genotype interaction effect between the TE/PTSD [χ ^2^ (2, *N*=149) = 8.91, *p*=.01] and TE/Control [χ ^2^ (2, *N*=190) = 4.72, *p*=.03] groups, but no significant interaction effect between the Control and PTSD groups (*p*=.36) In the TE group, Val/Met participants had a lower false alarm rate than Val/Vals. However, in the Control and PTSD groups, both had a higher false alarm rate for Val/Met than Val/Val. There was a main effect of genotype between the Control/PTSD groups in that Val/Mets had significantly higher false alarm rates than Val/Vals [χ ^2^ (2, *N*=129) = 5.30, *p*=.02, *d*=.50] but no significant group effect (*p*=.07).

*Figures 3****a*** & ***b***: Mean negative Hit rate scores for the Control, TE and PTSD groups and for the Val/Val and Val/Met Genotype groups. There was a significant main effect of group [χ ^2^ (2, *N*=234) = 6.21, *p*=.05] and genotype [χ ^2^ (1, *N*=234) = 5.34, *p*=.02], but no significant sex or interaction effects (all *p* >.69). PTSD participants had a significantly lower hit rate for negative images than Controls (*p*=.05, 95% CI [.00, .13]) with no significant hit rate differences between the PTSD and TE (*p*=.31, 95% CI [-.09, .03]) or TE and control groups (*p*=.12, 95% CI[-.08,.01]. Val/Val genotypes had a significantly higher hit rate (*m*=.86, *SD*=.11) than Val/Met participants (*M*=.82, *SD*=.12).

*Note*: Error bars: 95% CI

*Figure 2*. Mean d-prime scores for BDNF genotype across the PTSD, TE and control groups in Caucasian only participants

*Note*: Error bars: 95% CI
